# Irreducible Dorsal Distal Interphalangeal Joint Dislocation of Finger: A Case Report and Perspectives on Management

**DOI:** 10.7759/cureus.4588

**Published:** 2019-05-02

**Authors:** Sei Haw Sem, Muhammad Faiz Omar, Rashdeen Fazwi Muhammad Nawawi

**Affiliations:** 1 Orthopaedics, Hospital Kuala Lumpur, Kuala Lumpur, MYS; 2 Orthopaedics, Hospital Selayang, Selangor, MYS

**Keywords:** irreducible dorsal dislocation, distal interphlangeal joint, volar plate interposition

## Abstract

Irreducible closed dorsal dislocation of distal interphalangeal (DIP) joint of the finger is a rare injury. The causes of irreducibility of the DIP joint are volar plate interposition, entrapment of flexor digitorum profundus tendon behind the head of middle phalanx, and buttonholing of the middle phalanx head through the volar plate or flexor tendon. Open reduction with a volar approach is recommended with the advantages of better wound healing, ease of releasing entrapped structures, and possibilities of a volar plate, collateral ligaments, and/or flexor tendon repair. We report a case of irreducible dorsal dislocation of left ring finger DIP joint secondary to volar plate interposition treated successfully with open reduction.

## Introduction

Dislocations of the finger distal interphalangeal (DIP) joint are rare injuries due to its inherent stability provided by flexor and extensor tendon insertions, strong volar plate and collateral ligaments, and short lever arm of distal phalanx [1]. It is usually encountered in open injuries and reduction is easy in most of the cases with simple maneuver. We report a case of irreducible closed dorsal dislocation of the finger DIP joint to provide a better understanding of the underlying pathology and the rational of the surgical treatment.

## Case presentation

An 11-year-old boy presented to the emergency room with pain and swelling over his dominant left ring finger after a fall from a flight of stairs with no open wound. X-rays showed dorsal dislocation of the DIP joint of the left ring finger. Multiple attempts of closed reduction were unsuccessful and he was referred to the Hand and Microsurgery unit for further management, but the patient presented to our clinic only two weeks later. 

**Figure 1:**
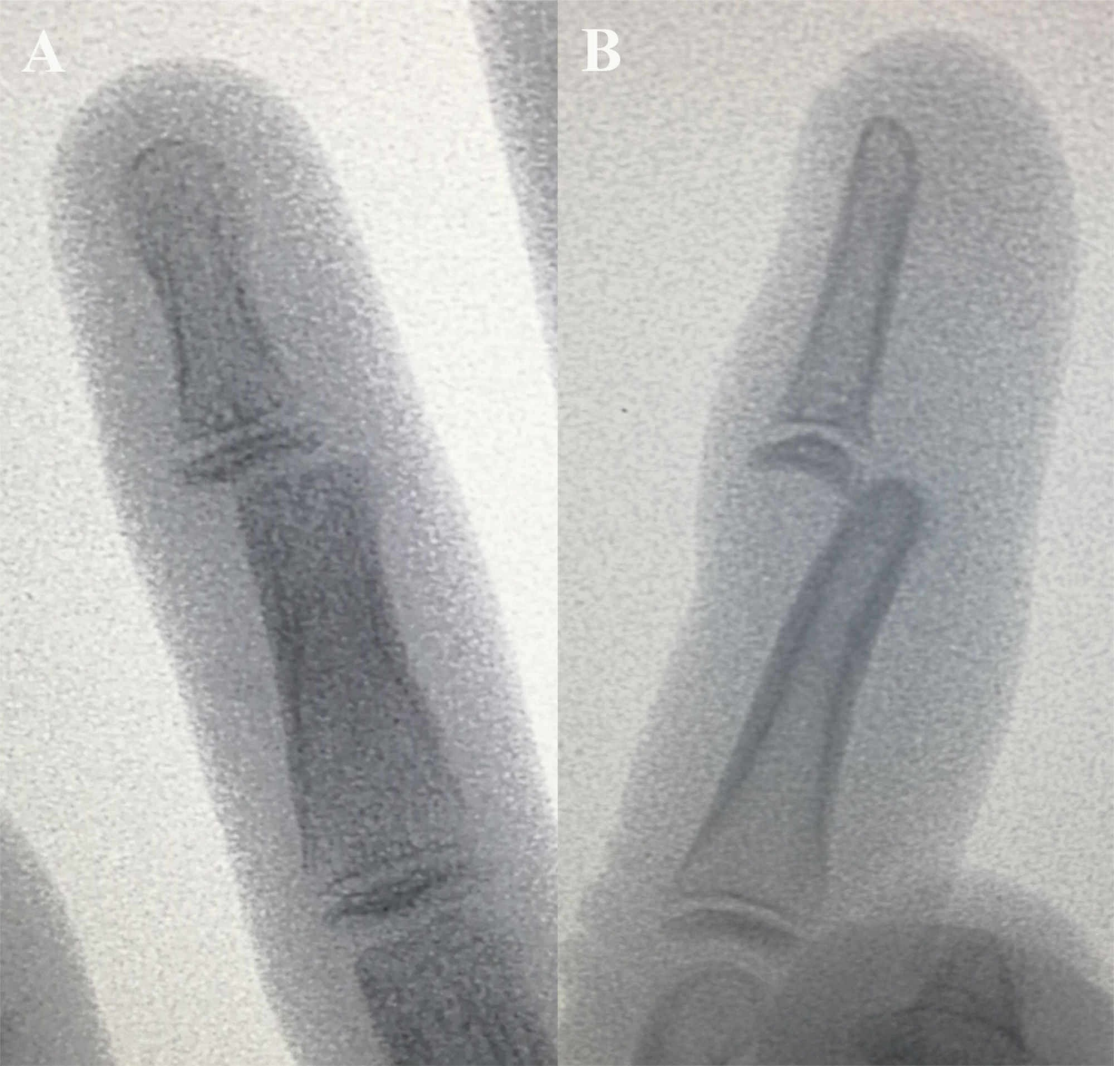
Preoperative radiographs revealed straight dorsal dislocation of the distal phalanx of the left ring finger in posteroanterior (A) and lateral (B) views

On examination, the DIP joint of left ring finger was swollen and tender. He was unable to flex or extend the DIP joint with normal movements over the metacarpophalangeal and proximal interphalangeal joints. Neurovascular status of the left ring finger was normal. Repeated radiographic assessment of the left ring finger revealed dorsal dislocation of the distal phalanx with no fracture seen (Figure 1). No further attempt of closed reduction was made and he was subjected for surgery after informed consent was obtained from his parents.

**Figure 2:**
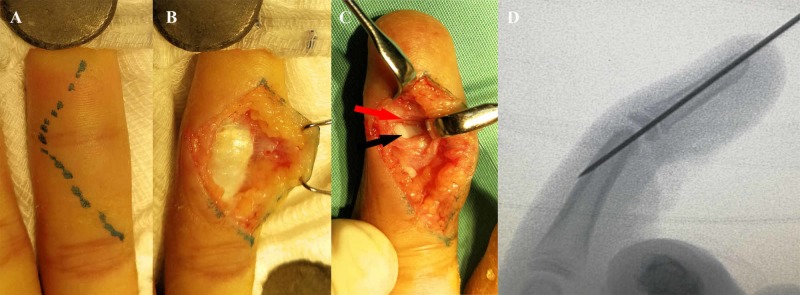
Surgical technique Volar approach was utilized for open reduction (A). Flexor digitorum profundus tendon was intact and not displaced (B). The volar plate (red arrow) was avulsed from its proximal attachment and interposed in between the head of middle phalanx (black arrow) and base of the distal phalanx (C). Post reduction, the distal interphalangeal joint was transfixed with k-wire in slight flexion (D).

He underwent open reduction and k-wire fixation of the left ring finger DIP joint via volar approach (Figure 2A). Intra-operatively, the flexor digitorum profundus (FDP) tendon was intact and not displaced (Figure 2B). Volar plate was avulsed from its proximal attachment and trapped in between the distal phalanx and head of the middle phalanx (Figure 2C). The DIP joint was reduced successfully after reposition of the volar plate. The volar plate was not repaired because the joint was stable, but it was immobilized in a slightly flexed position (10 to 15^o^) with a 0.039-inch K-wire (Figure 2D).

**Figure 3:**
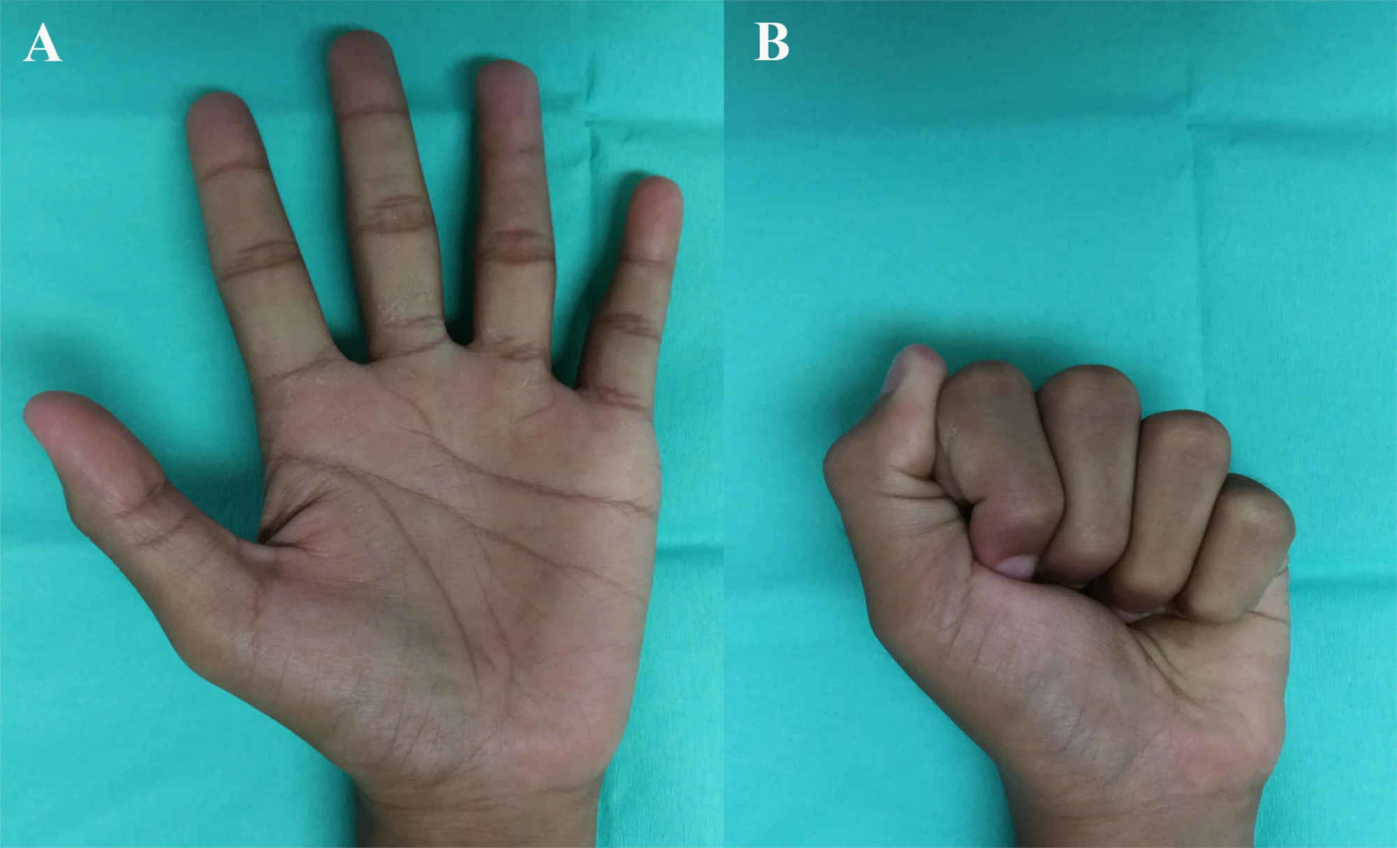
Postoperative assessment at 12 months Good functional range of motion of the left ring finger distal interphalangeal joint in extension (A) and flexion (B)

The K-wire was removed eight weeks after the surgery. Active and passive range of motion exercises were then started. Follow-up at 12 months revealed full range of motion of the left ring finger DIP joint with no residual pain or instability (Figure 3).

## Discussion

DIP joint is a hinge joint that only permits flexion and extension. Stability of the joint contributed minimally by the bony morphology but mainly by the surrounding ligamentous complexes. Volar plate, collateral ligaments, and FDP resist hyperextension, whereas extensor terminal tendon prevents hyperflexion of the joint [1]. Dislocations of the DIP joint are the result of combined axial compression and hyperflexion or hyperextension forces. Reduction of the dislocated joint usually happens spontaneously or with simple longitudinal traction. A maneuver with a combination of axial traction, hyperextension, and followed by direct pressure to the base of the distal phalanx is useful in the reduction of the dorsally dislocated DIP joint. Rarely, irreducible dislocations of the DIP joint are encountered. To date, there are not many cases reported in the literature with dorsal dislocations more common than volar dislocations [2].

There are four main causes for the irreducibility of the dislocated DIP joint, which are caused by FDP tendon and volar plate. The dislocated joint can be interposed by subluxated FDP tendon into the dorsum of one of the middle phalanx condyle or avulsed volar plate from the middle phalanx. Besides, buttonholing of the middle phalanx head through a split in the FDP tendon or volar plate can also be the underlying pathology [3]. There is difference between open and closed injuries with respect to the causes of irreducibility. In open dislocations, displacement of the FDP tendon dorsal to the middle phalanx condyle is the usual cause [4]. Avulsion and entrapment of the volar plate in the joint is commonly seen in closed injuries, as is found in this case.

Radiographic assessment is important in making the diagnosis and useful in understanding the underlying cause for the irreducibility. Straight dorsal dislocation in posteroanterior view of X-rays is suggestive of volar plate interposition, buttonholing through the volar plate or FDP tendon. Dorsal dislocation together with ulnar or radial dislocation of the distal phalanx indicates displacement of FDP dorsal to the middle phalanx condyle [5]. For this patient, the distal phalanx was dislocated dorsal-centrally and intra-operative finding was avulsion and interposition of the volar plate between two phalanges. 

Controversy exists with regard to the surgical approach in open reduction for this type of injury. The dorsal approach enables the joint to be assessed and reduced easily, but it creates a risk for wound healing and zero visibility of the volar structures. We chose the volar approach because it allows excellent visualization of the entrapped structure, assessment of associated injuries such as FDP tendon rupture and collateral ligaments, and good repair of the injured structures as needed [4]. In this case, only the volar plate was avulsed and entrapped.

Immobilization with k wire is not mandatory if the joint is stable post reduction [2]. For this patient, the DIP joint was stable after reduction but we still immobilized it with k* *wire in slight flexion to allow healing of the volar plate and we were cautious with the patient’s postoperative compliance. Generally, four weeks of immobilization for the DIP joint postoperatively is adequate and a shorter period is advisable for pediatric age group patients. However, the k wire was removed late in this case (eight weeks after surgery) because of the patient's compliance with the follow- up. Nonetheless, he regained good functional outcome with a full range of motion for the left ring finger DIP joint. 

## Conclusions

In conclusion, isolated closed dorsal dislocations of DIP joint are easily missed and commonly mismanaged. Without proper management, it often leads to chronic pain, stiffness, and poor hand function. Correct surgical decision and great functional outcomes require thorough understandings of the anatomy and pathology of the dislocated DIP joint.
